# Supplementary material for: The WAVE Regulatory Complex Is Required to Balance Protrusion and Adhesion in Migration
Source: Cells. 2020 Jul 7;9(7):1635. doi: 10.3390/cells9071635 (PMC7407199; doi:10.3390/cells9071635)
Supplement: Supplementary file 1 [file cells-09-01635-s001.zip › cells-837410_Supplementary material/cells-837410-Supplementary Material-Figures.pdf]

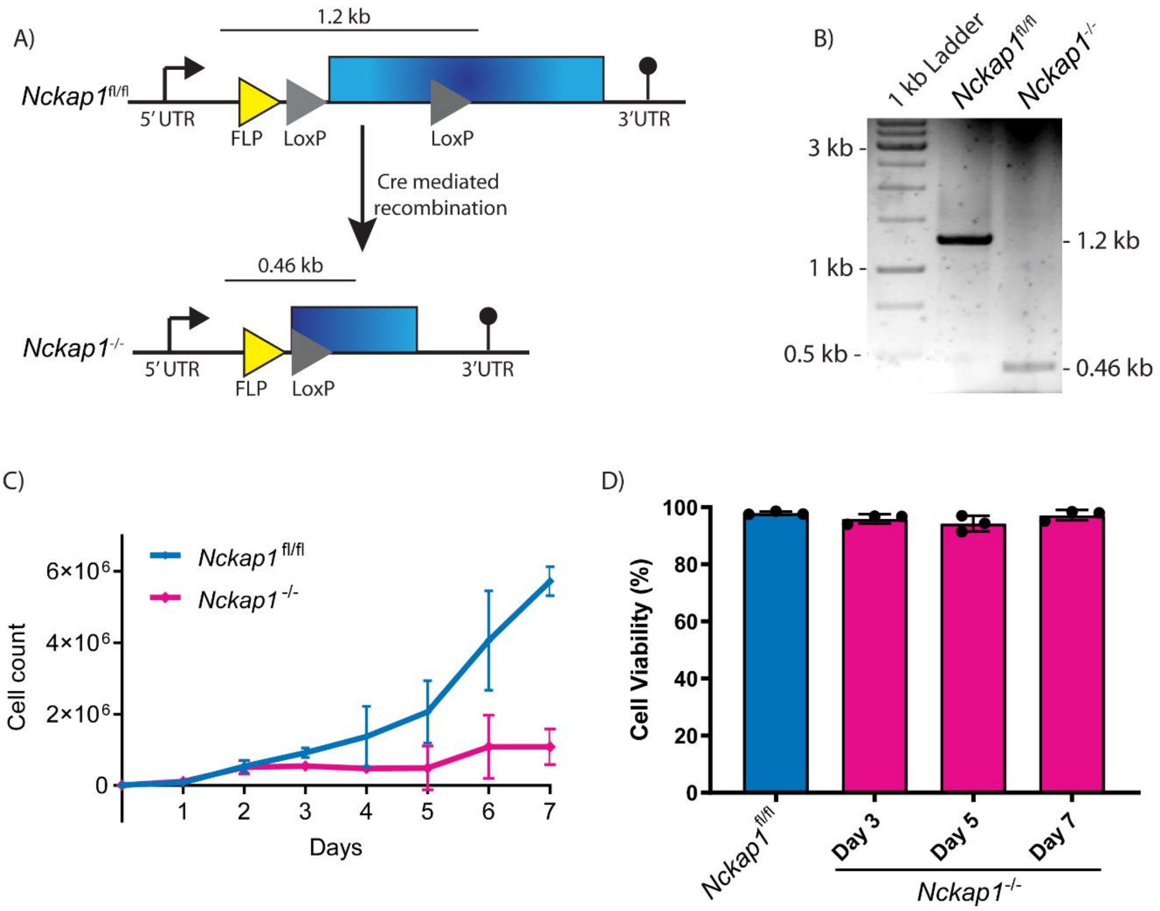

Figure S1. Inducible *Nckap1* KO MEFs, proliferation and viability. (**A**) Schematic of inducible *Nckap1*$^{fl/fl}$ gene in mouse embryonic fibroblasts. Upon Cre-mediated recombination the N-terminal region of *Nckap1* is deleted creating *Nckap1*$^{-/-}$ MEFs. (**B**) Analytical PCR after OHT treatment for 5 days. PCR product alters from a 1.2 kb to a 0.46 kb fragment, depicted in (**A**). (**D**) Cell proliferation assay. Harvested cells counted each day and displayed as an average from 3 independent experiments. Error bars represent S.D. (**E**) Cell viability determined through trypan blue solution presented as a percentage. *Nckap1* KO cells were harvested 3, 5 and 7 days post OHT treatment and counted. n = 3 independent experiments, error bars represent S.D.

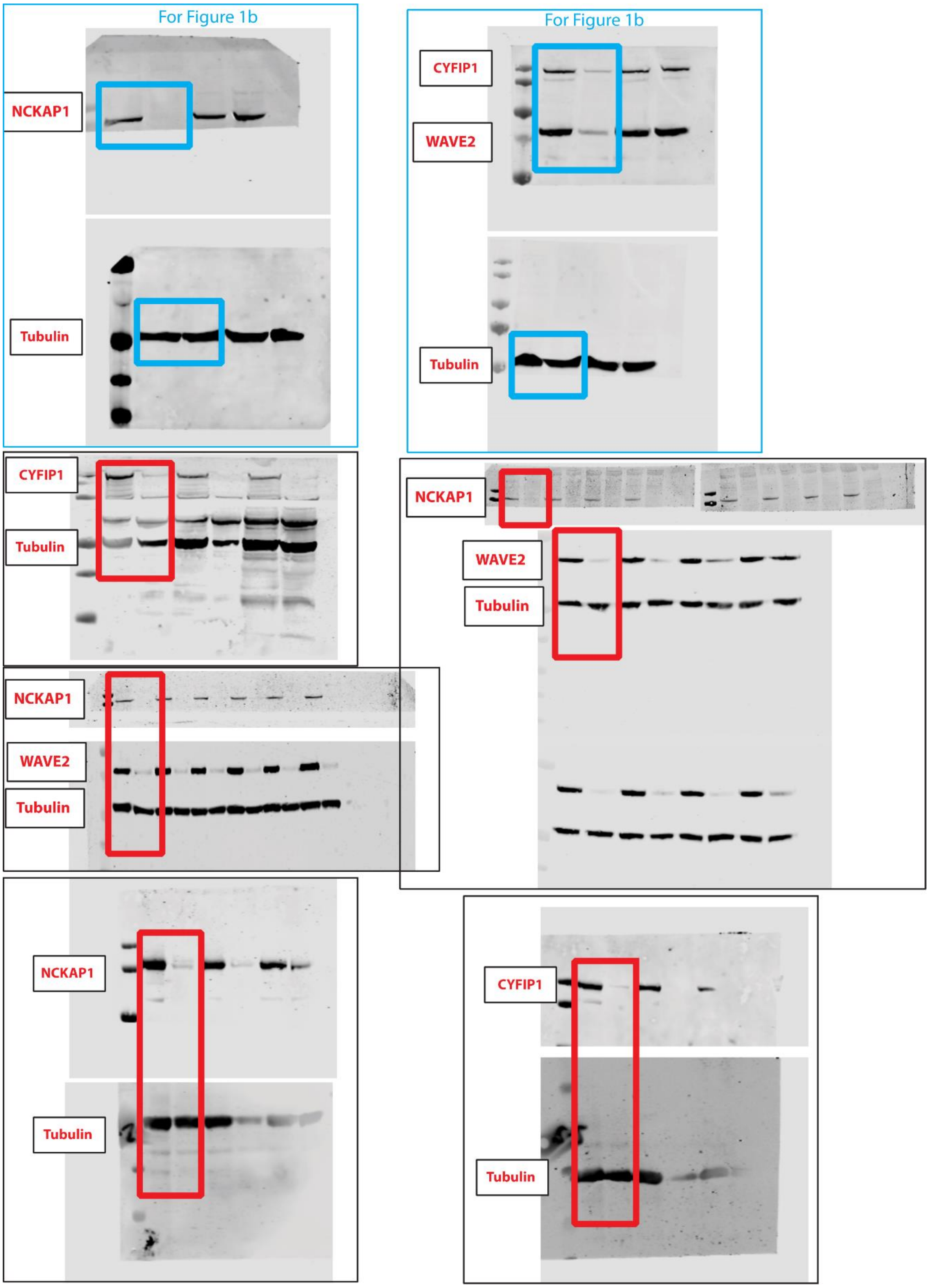

Figure S2. Raw Western blot images. NCKAP1 was stained for on 4 blots, WAVE2 on three blots and CYFIP1 on three blots. Tubulin loading controls depicted. Blue boxes indicate the blots used for Figure 1b.
